# Effect of Omega-3 fatty acids supplementation on serum level of C-reactive protein in patients with COVID-19: a systematic review and meta-analysis of randomized controlled trials

**DOI:** 10.1186/s12967-022-03604-3

**Published:** 2022-09-05

**Authors:** Amira Mohamed Taha, Ahmad Shehata Shaarawy, Mohamed Mosad Omar, Khaled Abouelmagd, Noran Magdy Shalma, Mais Alhashemi, Hala Mahmoud Ahmed, Ahmed Hafez Allam, Mohamed Abd-ElGawad

**Affiliations:** 1 Faculty of Medicine, Fayoum University, Postal address; Bank street, Talat, Fayoum, 63622 Egypt; 2 Faculty of Medicine, Al-Azhar University, Cairo, Egypt; 3 Kasr Alainy School of Medicine, Cairo University, Cairo, Egypt; 4 Cardiology Department, Al-Azhar University, New Damietta, Egypt; 5 Faculty of Medicine, Tanta University, Tanta, Egypt; 6 Faculty of Medicine, University of Aleppo, Aleppo, Syria; 7 Faculty of Medicine, Zagazig University, Sharkia, Egypt; 8 Faculty of Medicine, Menoufia University, Shebin El-Kom, Menoufia, Egypt; 9 Faculty of Medicine, Fayoum University, Fayoum, Egypt

**Keywords:** Omega-3, COVID-19, CRP

## Abstract

**Background:**

Omega-3 may alleviate the severity of coronavirus disease 2019 (COVID-19) by reducing the C-reactive protein (CRP) level, a marker for systemic inflammation. Because the scientific evidence indicating such a role is inconsistent, we aimed to evaluate the effect of Omega-3 on CRP change and CRP level in patients with COVID-19.

**Methods:**

We conducted a comprehensive search on four databases (PubMed, Web of Science, EMBASE, and Scopus). We included all RCTs comparing Omega-3 with a control group regarding their effect on the CRP levels in patients with COVID-19. We used version two of the Cochrane risk of bias assessment tool to appraise the included studies. We extracted data to an online data extraction sheet. The primary outcomes were CRP change from baseline and CRP serum levels.

**Results:**

We included four randomized controlled trials (RCTs) with 274 patients in this study. The overall effect estimate favored Omega-3 over the control group in terms of CRP change from baseline (mean difference (MD) =− 2.53, 95% confidence interval (CI): − 4.40, − 0.66) and CRP serum levels at the end of the study (MD =− 6.24, 95% CI: − 11.93, − 0.54).

**Conclusion:**

Omega-3 showed promising effects on systemic inflammation by reducing CRP levels in COVID-19 patients. Based on this finding, we recommend Omega-3 for COVID-19 patients for its anti-inflammatory actions.

**Supplementary Information:**

The online version contains supplementary material available at 10.1186/s12967-022-03604-3.

## Introduction

The coronavirus disease 2019 (COVID-19) pandemic has caused significant morbidity and mortality, engrossing the biomedical community in efforts to understand the pathophysiology of the disease and identify the most effective therapeutic strategies [1]. The World Health Organization (WHO) reported that until April 2022, COVID-19 global deaths had reached 6.21 million. In affected patients, COVID-19 generally induces a predictable pattern of metabolic and clinical disturbances [2]. It causes changes in their leukocyte counts, an increase in cytokines and inflammatory markers like C- reactive protein (CRP), and erythrocyte sedimentation rate (ESR). The higher the serum levels of inflammatory markers, the greater the severity of the disease [3, 4].

Nutritional deficiencies worsen both the immune response and viral pathogenic functions. As a result, oxidative stress may occur, leading to changes in the virus's genome, converting a normally benign or mildly pathogenic virus to a highly virulent pathogen [5, 6]. The pathogenicity of COVID-19 indicates that an adequate nutritional status is crucial for both early viremic and later hyperinflammatory stages [7].

Symptomatic COVID-19 patients have modest therapeutic modalities for alleviating their symptoms, decreasing inflammation, and slowing disease progression [8]. Furthermore, the effective drugs were evaluated in patients who were hospitalized or seriously ill [9]. As so, nutritional supplementations could modulate this inflammatory cascade and lead to better outcomes [10].

COVID-19 patients may develop acute respiratory distress syndrome (ARDS) due to the uncontrolled production of inflammatory mediators [11]. This inflammatory cascade may be modulated by nutritional supplements [10]. Nutritional supplements, including micronutrients such as; selenium (Se), zinc (Zn), and iron (Fe), are crucial for immune modulation in viral diseases, such as; chronic hepatitis and COVID-19 [12, 13]. Most COVID-19 patients exhibit combined Zn and Se deficiency [14]. Zinc can limit cytokine storm as well as the replication of the COVID-19 virus [15]. A recent study demonstrated a statistically significant association between the serum Se level and the cure rate in COVID-19 patients [16]. Se biomarkers were associated with decreased inflammatory markers, mainly CRP [17].

International nutrition associations recommended Omega-3 fatty acid-rich treatments to boost the immunological response in COVID-19 patients [18, 19].

Omega-3 polyunsaturated fatty acids (n-3 PUFAs) have become popular in nutrition for their beneficial usage in inflammatory diseases. Omega-3 fatty acids, such as eicosapentaenoic acid (EPA) and docosahexaenoic acid (DHA), are long-chain n-3 PUFAs that are known for their promising anti-inflammatory roles against inflammatory diseases [20].

Previous studies on diabetes, sepsis,, and hemodialysis patients have demonstrated the ability of Omega-3 supplementations to decrease CRP and other inflammatory markers. Mortality in COVID-19 patients is considerably elevated with associated co-morbidities [21]. Diabetic patients are more likely to experience abnormal clotting and thrombus formation exacerbated by COVID-19 [22, 23].

Omega-3 PUFAs can reduce inflammation through different mechanisms. They decrease the production of arachidonic acid-derived eicosanoid mediators, which have pro-inflammatory properties [24]. Moreover, the enzymatic oxidation of EPA and DHA produces resolvins and protectins, which aid in the resolution of inflammation. In vitro and in vivo studies revealed that such a mechanism could need Omega-3 fatty acid concentrations as high as 3 g. [25]

In the recent REDUCE-IT trial, icosapent ethyl (IPE), which is a synthetic derivative of EPA (at an oral dose of 4 g daily), was associated with a 25% reduction in cardiovascular complications in patients with or at risk of cardiovascular disease over a 4.9-year median follow-up period [8]. Another published trial by Doaei et al. used a daily 1000 mg concoction (200 mg DHA and 400 mg EPA) for a two-week follow-up period as a therapeutic agent against COVID-19. The study showed that the one-month survival rates, renal functions, and impacts on arterial blood gas indicators were higher in the treatment group compared to the control group [26].

Given the positive impact of Omega 3 fatty acids on the inflammatory process, the rising use of Omega-3 PUFAs as immune nutrients for COVID-19 patients, and the contradictory findings of recent trials on the effect of Omega-3 supplementations on inflammatory biomarkers in COVID-19 patients, we aimed to assess the impact of Omega-3 PUFAs supplementations on the CRP levels in COVID-19 patients.

## Methods

We followed the Preferred Reporting Items for Systematic Reviews and Meta-Analyses (PRISMA) statement during the preparation of this meta-analysis [27]. The PRISMA Checklist is included in Additional file 1*.*

### Inclusion and exclusion criteria

We included all randomized controlled trials (RCTs) satisfying the following criteria: (1) studies conducted on patients diagnosed with COVID-19; (2) Studies that used Omega-3 PUFAs or any other derivatives as their primary intervention, regardless of dose and form of administration, and whether alone or in addition to other drugs; (3) Studies that reported CRP levels as one of their outcomes. We excluded articles not satisfying these criteria. We also excluded abstracts, expert opinions, editorials, animal studies, letters, and studies unavailable in English.

### Literature search

We applied a search strategy to four electronic databases: PubMed, Scopus, Web of Science, and EMBASE in March 2022. Relevant articles were identified using the following search strategy: ("Omega-3 Fatty Acid" OR "n3 Oil" OR "n3 Fatty Acid" OR "n3 PUFA" OR "n3 Polyunsaturated Fatty Acid" OR "N 3 Fatty Acid" OR "n 3 Polyunsaturated Fatty Acid") AND ("COVID 19" OR "SARS-CoV-2 Infection" OR "2019 Novel Coronavirus Disease" OR "2019 Novel Coronavirus Infection" OR "COVID 19 Virus Infection" OR "Coronavirus Disease 2019" OR "Severe Acute Respiratory Syndrome Coronavirus 2 Infection" OR "SARS Coronavirus 2 Infection" OR "COVID 19 Virus Disease" OR "2019 nCoV Infection" OR "COVID-19 Pandemics"). Detailed search strategies for all four databases are elucidated in Additional file 2.

### Study selection

All authors independently screened the retrieved studies for eligibility in two phases. First, titles and abstracts were screened for eligibility. Then, a full-text screening of the eligible abstracts was performed to select the final included studies in the meta-analysis.

### Quality assessment

Two authors independently performed a quality assessment of the retrieved RCTs. A senior author resolved any disagreements. The evaluation was done according to version 2 of the Cochrane tool for assessing the risk of bias in randomized trials (RoB2) [28], which assesses the following items: randomization process, deviations from intended interventions, missing outcome data, measurement of the outcome and selection of the reported result. We judged each study as either low or high risk of bias or some concerns. We used robvis to create the risk-of-bias plots [29].

### Data extraction

Two authors independently extracted the following data using an online data extraction sheet: (1) summary of the included studies (study design, study location, sample size, population, interventional regimens, control, follow-up period, and primary outcomes); (2) Baseline characteristics of the included participants (age, gender, body mass index (BMI), and baseline CRP levels); and (3) study outcomes (CRP levels).

### Statistical analysis

Review Manager (RevMan) version 5.4 for Windows was used to perform the statistical analysis. Significant results were defined with a P-value of less than 0.05. Data were pooled as mean difference (MD). Initially, we used the fixed effect model, assuming that the studies were homogenous due to the similarity in study design and treatment effect measures. Random-effects meta-analysis model was used to pool heterogeneous data using the Mantel–Haenszel equation. We evaluated statistical heterogeneity by visual inspection of the forest plots. We also measured its magnitude by the I-square and Chi-Square tests. Significant heterogeneity was detected when the chi-square P-value was less than 0.05. The I-square test was employed to measure the extent of heterogeneity according to the Cochrane Handbook of Systematic reviews and meta-analysis recommendations. I-square more than 75% was considered a significant heterogeneity[30].

## Results

### Search results

**Fig. 1:**
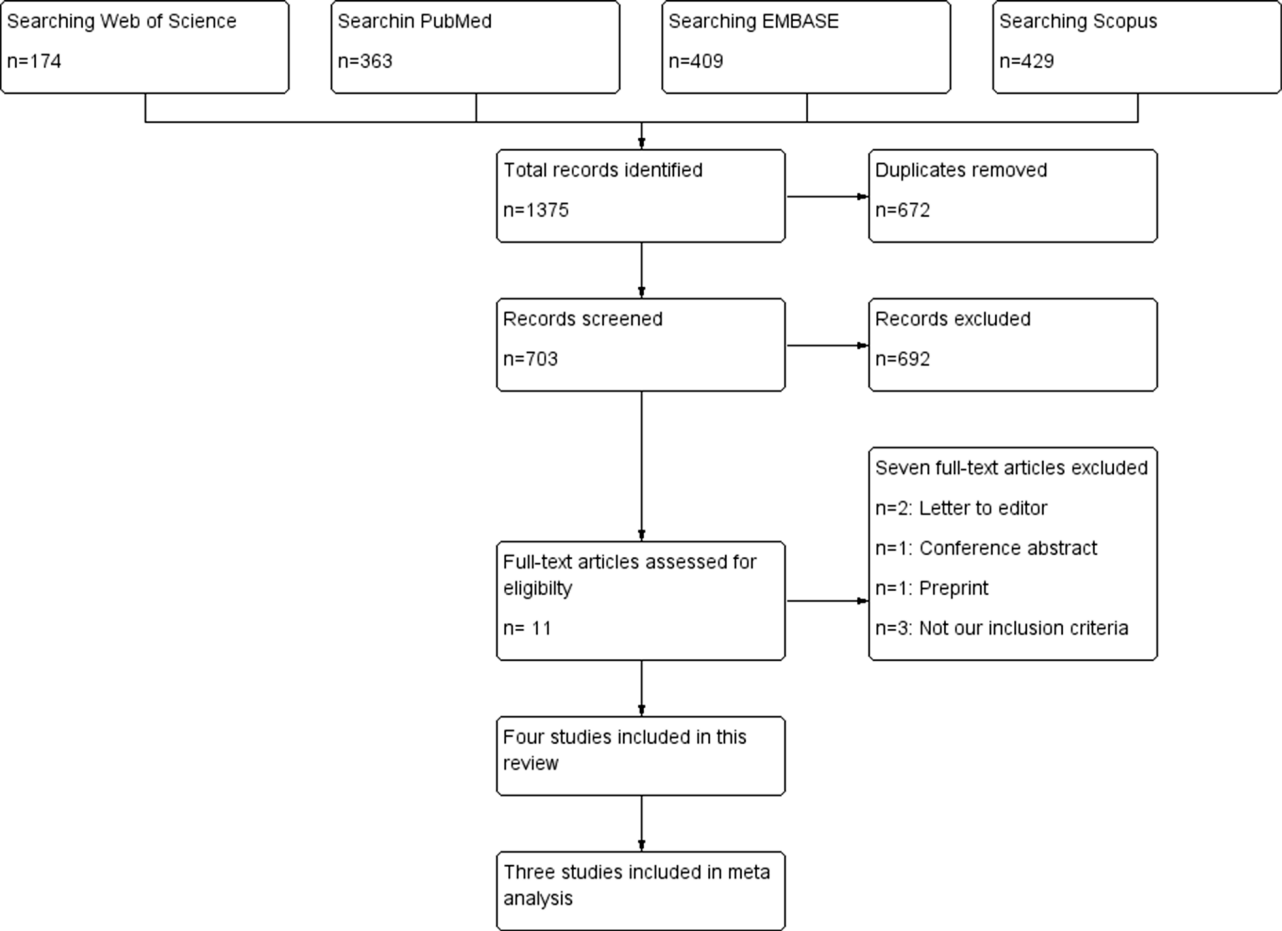
PRISMA flow diagram

Our literature search strategy retrieved 1375 records. We found 11 eligible articles for full-text screening after evaluating titles and abstracts. Of them, four studies were included in this systematic review. However, only three studies were pooled in the meta-analysis, as shown in Fig. 1.

### Characteristics of the included studies

We included four studies [26, 31–33] in this meta-analysis, totaling 274 COVID-19 patients. Patients were randomly assigned to receive Omega-3 or other derivatives as an intervention or control in all trials. A summary of the characteristics of the included studies is illustrated in Table 1. The baseline characteristics of their participants are shown in Table 2.

**Table 1:** Summary of included studies

Study ID	Design	Country	Sample size	Population & Inclusion criteria	Intervention	Control	Follow-up period (days)	Primary outcomes
Pimentel 2022	Double-blinded RCT	Brazil	43	Patients diagnosed with COVID-19 through molecular examination (RT-PCR), aged between 18 to 65 years old, fed with oral diet, with patent gastrointestinal tract, and not requiring ICU hospitalization or mechanical ventilation	200 ml units of a high-protein, normocaloric nutritional supplement enriched with L-arginine, nucleotides, and omega-3 essential fatty acids	200 ml units of a high protein, normocaloric nutritional supplement that did not include any additional immune nutrients	8	Changes in serum CRP level and total lymphocyte count
Sedighiyan 2021	Single-blinded RCT	Iran	30	Patients diagnosed with COVID-19, aged > 18, with BMI > 18.5	Omega-3 (3 capsules containing 670 mg EPA and DHA) + 2 capsules containing 400 mg Hydroxychloroquine	Two capsules containing 400 mg hydroxychloroquine	14	Changes in serum CRP, ESR, and clinical symptoms, including pain, fatigue, appetite, and olfaction
Kosmopoulos 2021	Open-label RCT	Canada	100	Patients diagnosed with COVID-19, with a positive PCR test result obtained within the previous 72 h of enrollment, having at least one of these symptoms: cough, fever, sore throat, myalgia, or shortness of breath	Icosapent ethyl (8 g daily for three days, followed by 4 g daily for 11 days)	Usual care (no intervention)	14 + 3	Change in high-sensitivity C-reactive protein (hs-CRP) and change in InFLUenza Patient-Reported Outcome (FLU-PRO) diary scores
Doaei 2021	Double-blind RCT	Iran	101	Patients aged between 35 and 85 years diagnosed with COVID-19 through a positive RT-PCR nasopharyngeal swab and with the following symptoms: fever, fatigue, dry cough, respiratory distress, or severe pneumonia. Patients also had to be indicated for enteral nutrition	One capsule of 1000 mg omega-3 daily containing 400 mg EPA and 200 mg DHA, through adding the supplement to their enteral formula	Placebo in the form of an isocaloric-isovolemic formula	14	Blood glucose, sodium (Na), potassium (K), blood urea nitrogen (BUN), creatinine (Cr), albumin, hematocrit (HCT), calcium (Ca), phosphorus (P), mean arterial pressure (MAP), O2 saturation (O2sat), arterial pH, partial pressure of oxygen (PO2), partial pressure of carbon dioxide (PCO2) and bicarbonate (HCO3), as well as 1-month survival rate

*RT*-*PCR* reverse transcription-polymerase chain reaction, *CRP* C-reactive protein, *RCT* randomized controlled trial, *BMI* body mass index, *EPA* Eicosapentaenoic acid, *DHA* docosahexaenoic acid, *ESR* erythrocyte sedimentation rate, COVID-19 coronavirus disease 2019

**Table 2:** Baseline characteristics of included studies

Study ID	Study groups	Sample size	Age mean (SD)	Gender male (%)	Bmi mean (SD)
Pimentel 2022	Immunonutrient enriched supplement	21	41.1 (2.8)	14 (66.7)	27.3 (1.2)
	control group (non-immunonutrient enriched supplement)	22	41.9 (2.6)	12 (54.5)	27.8 (1)
Sedighiyan 2021	Omega-3 + hydroxychloroquine	15	66.46 (2.8)	9 (60)	27.01 (0.94)
	Control group (hydroxychloroquine alone)	15	67.06 (2.28)	9 (60)	26.13 (0.73)
Kosmopoulos 2021	Icosapent ethyl	50	44 (16.29)	24 (48)	24.83 (3.33)
	Control group	50	40.66 (17.77)	21 (42)	24.36 (4.22)
Doaei 2021	1000 mg omega-3	28	66 (14.58)	15 (53.6)	27.68 (7.54)
	Control group	73	64 (14.25)	45(61.6)	27.39 (3.15)

BMI: body mass index, *SD* standard deviation

Doaei et al. [26] conducted a double-blind RCT to evaluate the effect of Omega-3 PUFA supplementation on inflammatory and biochemical markers in COVID-19 patients who were critically ill. CRP level was one of the primary outcomes of this study; however, they failed to measure its level, which was considered a major limitation in their study.

### Quality assessment results

The overall risk of bias in the included studies [26, 31–33] was high.

Regarding the randomization process, two included studies [26, 32] reported an adequate randomization method with allocation concealment, so we judged them as low risk. However, Sedighiyan et al.[33] and Kosmopoulos et al. [31] were judged as some concerns.

Regarding deviations from the intended interventions, two studies; Pimentel et al. [32] and Doaei et al. [26] were judged as low risk of bias due to sufficient blinding of patients and examiners, while the other two studies: Kosmopoulos et al. [31] and Sedighiyan et al. [33] were judged as high risk as they were open-labeled and single-blinded studies, respectively.

Regarding missing outcome data, Kosmopoulos et al. [31]and Pimentel et al. [32]had a low risk of bias due to sufficient outcome data and the use of intention to treat (ITT) analysis. Sedighiyan et al. [33] and Doaei et al. [26] were judged as high risk of bias due to missing data without using the ITT method for analysis.

Regarding the measurement of the outcomes, Kosmopoulos et al. [31] and Sedighiyan et al. [33] were judged as high risk as certain outcomes were patient-reported, and patients were not blinded. The other two studies [26, 32] were judged as low risk.

**Fig. 2:**
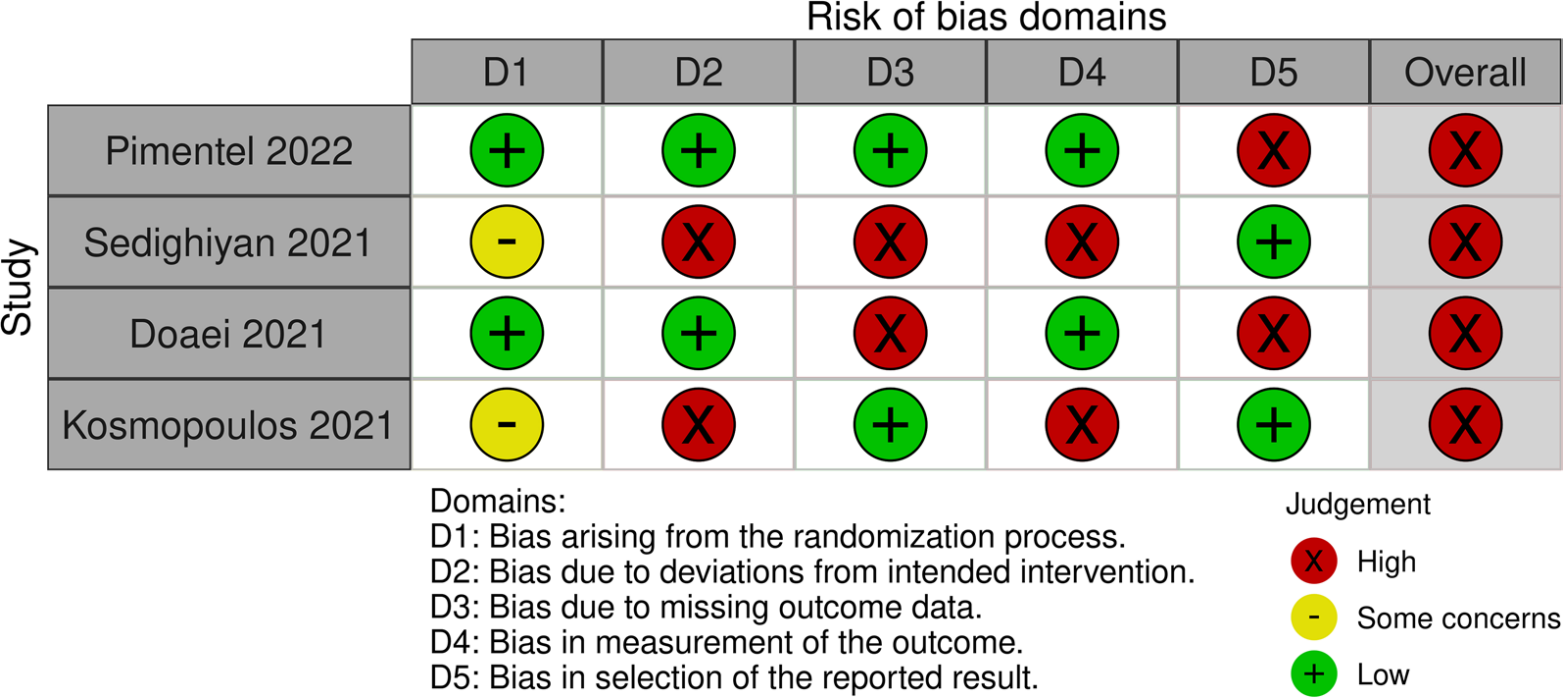
Risk of bias graph for included studies

**Fig. 3:**
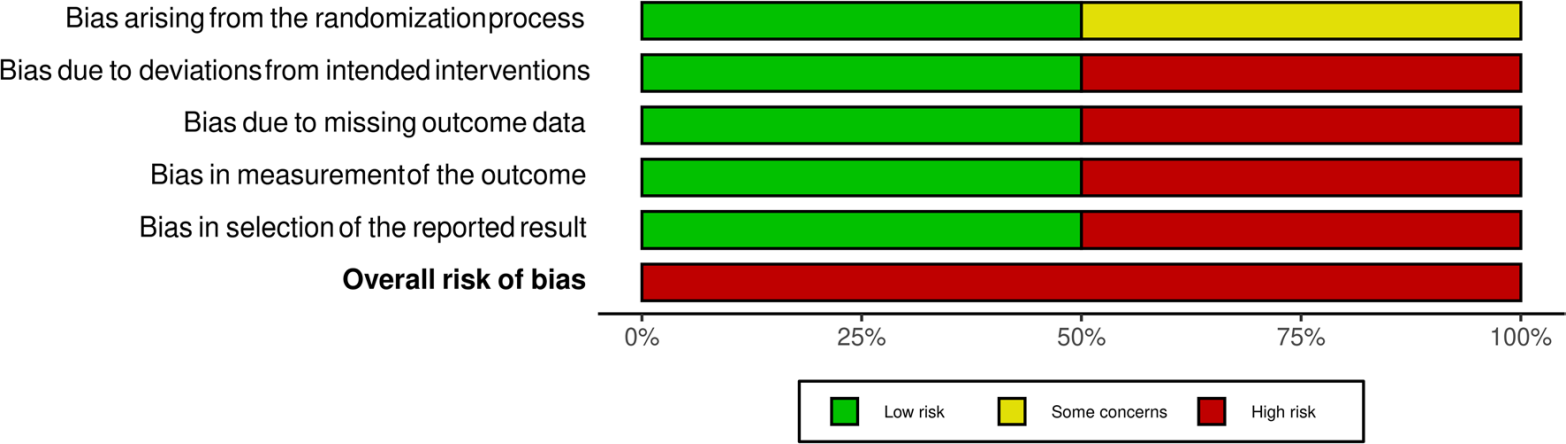
Risk of bias summary for included studies

As for bias in the selection of the reported results, Doaei et al. [26] and Pimentel et al. [32] were judged as high risk of bias due to incomplete outcomes reporting. On the other hand, Kosmopoulos et al. [31] and Sedighiyan et al. [33] were judged as low risk of bias due to reporting all outcomes in the protocol. Figures 2 and 3 illustrate a summary of the risk of bias assessment of the included studies.

### Outcomes

#### CRP serum levels

**Fig. 4:**
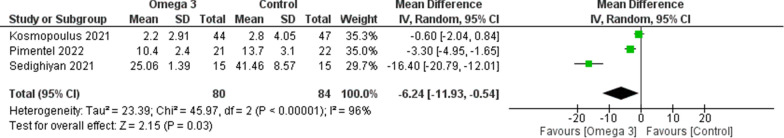
Forest plot of the C-reactive protein (CRP) serum levels

Pooled data from three studies [31–33] with 173 patients favored Omega-3 over the control group (MD =− 6.24; 95% CI: − 11.93, − 0.54; P = 0.03; Fig. 4) in terms of reducing the serum levels of CRP. Considerable heterogeneity was detected among these studies under a random-effects model (P > 0.00001, I^2^ = 96%).

#### CRP changes from baseline

**Fig. 5:**
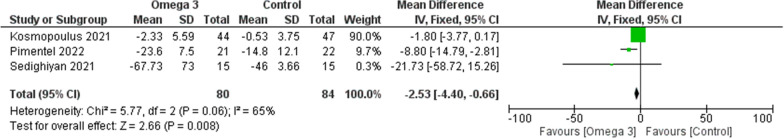
Forest plot of the change from baseline in C-reactive protein (CRP)

Pooled data from three studies [31–33] with 173 patients favored Omega-3 over the control group (MD =− 2.53; 95% CI: − 4.40, -0.66); P = 0.008; Fig. 5) in terms of the detected baseline change in CRP levels. Pooled data were homogenous under a fixed-effects model (P = 0.06, I^2^ = 65%).

## Discussion

This is, to our knowledge, the first systematic review and meta-analysis to assess the impact of Omega-3 fatty acid supplementation on the CRP levels of patients with COVID-19. Omega-3 fatty acids' beneficial effect in modulating the inflammatory process is well established in the literature [20, 24]. A decline in serum CRP level has been demonstrated following Omega-3 PUFAs supplementation in several diseases [34, 35]. However, inconsistent findings were observed in COVID-19 patients [26, 31–33]. To resolve this discrepancy, we conducted a systematic review of recently published RCTs by providing clear evidence regarding this debate.

The included trials in this systematic review assessed the effect of Omega-3 supplementation on the inflammatory biomarker CRP. Our results (three pooled studies with 173 patients) found a significant decrease in the CRP serum levels as an endpoint following Omega-3 supplementations in COVID-19 patients compared to the control. In addition, a significant change from baseline in the CRP levels was noted in our results favoring Omega-3 supplementations over the control.

Results from Kosmopoulos et al. [31], who studied the effects of IPE on ambulatory patients with COVID-19, indicate that the IPE group had reduced high-sensitivity CRP by 25% (p = 0.011) and improved symptoms compared to patients assigned to usual care who tended to have a non-significant reduction by 5.6% (p = 0.51). However, a non-significant result was obtained when comparing the two groups for unadjusted values (p = 0.082).

The concentrations of CRP in serum posttreatment were found to be significantly lower (p < 0.05) in the treatment group who took Omega-3 supplements compared to the baseline in the study by Sedighiyan et al. [33]. Furthermore, the treatment group's CRP levels were significantly reduced (p < 0.001). In 2021, Pimentel et al. [32] published a randomized clinical trial on 43 adult patients with COVID-19, which showed a significant CRP level reduction in the experiment group compared to the control group (p = 0.002).

This meta-analysis resolved this ongoing debate and highlighted the beneficial role of Omega-3 fatty acids administration on the CRP levels and the change in its levels from baseline. This could be explained by interleukin-6 (IL-6) which is a sensitive indicator in inflammatory conditions [36] as it induces the synthesis of CRP during inflammation, and it was significantly higher in severe COVID-19 cases [37]. N-3 PUFAs have decreased endotoxin-stimulated production of IL-6 and tumor necrosis factor (TNF)-α [20].

In a recently published RCT by Doaei et al. [26], critically ill COVID-19 patients were randomized to receive enteral omega-3 fatty acids or control for 14 days. Authors reported that patients supplemented with omega-3 showed improvement in respiratory and renal functions as indicated by significantly low BUN, Cr, and K levels and high pH and HCO3. This might be correlated to the effect of this supplementation in modulating the inflammatory cytokines.

Similarly, Kosmopoulos et al. [31] found that the IPE group scored significantly lower than the usual care cohort in the total systemic and respiratory symptoms among patients with COVID-19. However, the gastrointestinal domain had a considerable score decline in the standard care group compared to the IPE group. Additionally, Sadighiyan et al. [33] observed that short-term Omega-3 supplementations improved the clinical signs of COVID-19 infection compared to the control group, except for the olfactory impairment. Our study confirms these positive findings as the authors hypothesized that lower CRP, the main outcome of our meta-analysis, might be one of the reasons Omega-3 decreased COVID-19 physical symptoms as the improvements in inflammatory parameters are correlated to the clinical changes [33].

Infection with COVID-19 causes a "cytokine storm" similar to ARDS, resulting in systemic inflammation and multiple organ failure [3, 38, 39]. Recent research has shown that n-3 PUFAs such as EPA and DHA can regulate the immune response, reduce hyperinflammatory conditions, and decrease the complications of infection [40]. In patients with ARDS, a meta-analysis by Pontes-Arruda et al. [41] reported a significant decline in ventilator-free days, organ failures, length of stay in the intensive care unit (ICU), and mortality following EPA and gamma-linolenic acid (GLA) supplements. Langlois et al. found that patients with ARDS who received Omega-3 PUFAs supplements had decreased stay in the ICU and decreased duration of mechanical ventilation [42]. All these positive outcomes adhere to the reduction in CRP levels being a main marker of inflammation. Thus, this highlights the major rule of Omega-3 fatty acids in alleviating the cytokine storm of COVID-19 and all its associated co-morbidities.

After demonstrating that supplementation with N-3 PUFAs benefits CRP levels in this study, Insights for future research have emerged. Additional scientific evidence in controlling the inflammatory cascade could help alleviate COVID-19's damaging consequences on global health by reducing economic burdens.

### Strengths and limitations

We followed the PRISMA checklist and the Cochrane Handbook of systematic reviews in all performed steps. Strengths can be summarized as including only RCTs and strict inclusion and exclusion criteria that were required to reduce heterogeneity. However, this resulted in the main limitation of this study, which is including a few studies. Multiple variables limit the interpretation of the included trials, such as the small sample size and short follow-up period. In addition, Included studies are of limited quality and one of them provided Omega-3 and other supplements, which might have weakened its quality of evidence. Furthermore, our patients had various degrees of disease severity with different underlying comorbidities.

Three of the included studies [26, 31, 32] reported a non-significant increase in the lymphocyte count. However, the data represented were insufficient to be pooled in the meta-analysis. Furthermore, only two studies [31, 33] reported the ESR levels, which impeded our ability to make conclusions about this important biomarker.

## Conclusion

Based on this meta-analysis, Omega-3 supplementations showed significant benefits in alleviating the inflammatory response as indicated by the CRP level reduction. Thus, Omega-3 might be prescribed as a supplement for COVID-19 patients. These results seem promising; however, further RCTs with larger sample sizes and longer follow-up duration are needed to confirm our findings, considering the supplementation dose, follow-up duration, and patients' characteristics.

## Supplementary Information

**Additional file 1:** PRISMA Checklist.

**Additional file 2:** Search strategy.

## Data Availability

All data generated or analyzed during this study are included in this published article or the data repositories listed in References.

## Abbreviations

CRP: C-reactive protein
COVID-19: Coronavirus disease -19
RCTs: Randomized clinical trails
MD: Mean difference
CI: Confidence interval
WHO: World health organization
ESR: Erythrocyte sedimentation rate
ARDS: Acute respiratory distress syndrome
Se: Selenium
Zn: Zinc
Fe: Iron
n-3 PUFAs: Omega-3 polyunsaturated fatty acids
EPA: Eicosapentaenoic acid
DHA: Docosahexaenoic aci
IPA: Icosapent ethyl
PRISMA: Preferred reporting items for systematic reviews and meta-analyses
BMI: Body mass index
RevMan: Review manager
ITT: Intention to treat
IL-6: Interleukin-6
TNF-α: Tumor necrosis factor- α
BUN: Blood urea nitrogen
Cr: Creatinine
K levels: Potassium levels
PH: Potential of hydrogen
HCO3: Bicarbonate
ICU: Intensive care unit
GLA: Gamma linolenic acid
