# Supplementary material for: Effect of Omega-3 fatty acids supplementation on serum level of C-reactive protein in patients with COVID-19: a systematic review and meta-analysis of randomized controlled trials
Source: J Transl Med. 2022 Sep 5;20:401. doi: 10.1186/s12967-022-03604-3 (PMC9444081; doi:10.1186/s12967-022-03604-3)
Supplement: Supplementary file 2 — Additional file 2: Search strategy. [file 12967_2022_3604_MOESM2_ESM.docx]

**Effect of Omega-3 fatty acids supplementation on serum level of C-reactive protein in patients with COVID-19: A Systematic Review and Meta-Analysis of Randomized Controlled Trials**

Amira Mohamed Taha^1*^, Ahmad Shehata Shaarawy^2^, Mohamed Mosad Omar^3^, Khaled Abouelmagd^4^, Noran Magdy Shalma^5^, Mais Alhashemi^6^, Hala Mahmoud Ahmed^7^, Ahmed Hafez Allam^8^, Mohamed Abd-ElGawad^9^

^1*^ Faculty of Medicine, Fayoum University, Fayoum, Egypt

[am7529@fayoum.edu.eg](mailto:mohammed.mahmod87@gmail.com)

^2^ Faculty of Medicine, Al-Azhar University, Cairo, Egypt

AhmadShaarawy.2020@azhar.edu.eg

^3^ Kasr Alainy School of Medicine, Cairo University, Cairo, Egypt

[Mohamed-m-abdelmohsen@students.kasralainy.edu.eg](mailto:Mohamed-m-abdelmohsen@students.kasralainy.edu.eg)

^4^ Cardiology Department, Al-Azhar University, New Damietta, Egypt

[Khaled-Abouelmagd@domazhermedicine.edu.eg](mailto:Khaled-Abouelmagd@domazhermedicine.edu.eg)

^5^ Faculty of Medicine, Tanta University, Tanta, Egypt.

[Nouran_30942456@med.tanta.edu.eg](mailto:Nouran_30942456@med.tanta.edu.eg)

^6^ Faculty of Medicine, University of Aleppo, Aleppo, Syria

[alhashemi.mais@gmail.com](mailto:alhashemi.mais@gmail.com)

^7^ Faculty of Medicine, Zagazig University, Sharkia, Egypt

[20512018200519@medicine.zu.edu.eg](mailto:20512018200519@medicine.zu.edu.eg)

^8^ Faculty of Medicine, Menoufia University, Shebin El-Kom, Menoufia, Egypt

[ahmedhafez21@med.menofia.edu.eg](mailto:ahmedhafez21@med.menofia.edu.eg)

^9^ Faculty of Medicine, Fayoum University, Fayoum, Egypt

[mohammed.mahmod87@gmail.com](mailto:mohammed.mahmod87@gmail.com)

**Corresponding author***:

Amira Mohamed Taha,

Faculty of Medicine, Fayoum University, Fayoum, Egypt,

Postal address; Bank street, Talat, Fayoum, Fayoum, Egypt

Postal code: 63622

Tel. /Fax: +201017110154

Email: [am7529@fayoum.edu.eg](mailto:mohammed.mahmod87@gmail.com)

ORCID-ID:0000-0002-9620-7350

**Search strategy:**

***PUBMED:***

1. “Omega-3 Fatty Acid”[All Fields] OR “n3 Oil”[All Fields] OR “n3 Fatty Acid”[All Fields] OR “n3 PUFA”[All Fields] OR “n3 Polyunsaturated Fatty Acid”[All Fields] OR “N 3 Fatty Acid”[All Fields] OR n 3 Polyunsaturated Fatty Acid OR "Fatty Acids, Omega-3"[Mesh] OR “Fish Oil”[All Fields] OR “Fish Liver Oils”[All Fields] OR "Fish Oils"[Mesh] OR “Linolenate”[All Fields] OR “alpha Linolenic Acid”[All Fields] OR “Linolenic Acid”[All Fields] OR “Omacor”[All Fields] OR “Lovaza”[All Fields] OR “EPA”[All Fields] OR “eicosapentaenoate-lipoate”[All Fields] OR “eicosapentaenoate-lipoate”[All Fields] OR “EPA-TA”[All Fields] OR “EPA-LA”[All Fields] OR “IPE”[All Fields] OR “Vascazen”[All Fields] OR “EPA-TA”[All Fields] OR “EPA-LA”[All Fields] OR “omega-3 ethyl ester 90”[All Fields] OR “P-OM3 adjunct”[All Fields] OR “ethyl-EPA”[All Fields] OR “ethyl eicosapentaenoate”[All Fields] OR “ethyl icosapentaenoate”[All Fields] OR “ethyl eicosapentaenoic acid”[All Fields] OR “icosapent ethyl”[All Fields] OR “IPE”[All Fields] OR “Epadel”[All Fields] OR “AMR101”[All Fields] OR vascepa”[All Fields] OR “Docosahexaenoic Acid”[All Fields] OR DHA”[All Fields] OR “Omegaven”[All Fields] OR “omtryg”[All Fields] OR Icosapent”[All Fields] OR “Doconexent”[All Fields] OR “Omega-3-carboxylic acids”[All Fields] OR “Resolvin E1”[All Fields] OR “Icosapent ethyl”[All Fields] OR Icosapent”[All Fields])
2. “COVID 19”[All Fields] OR “SARS-CoV-2 Infection”[All Fields] OR “2019 Novel Coronavirus Disease”[All Fields] OR “2019 Novel Coronavirus Infection”[All Fields] OR “COVID 19 Virus Infection”[All Fields] OR “Coronavirus Disease 2019”[All Fields] OR “Severe Acute Respiratory Syndrome Coronavirus 2 Infection”[All Fields] OR “SARS Coronavirus 2 Infection”[All Fields] OR “COVID 19 Virus Disease”[All Fields] OR “2019 nCoV Infection”[All Fields] OR “COVID-19 Pandemics”[All Fields] OR "COVID-19"[Mesh]
3. #1 AND #2

*Date of search: 10 March 2022*

*Search results: 363*

*No limitations applied*

***WEB OF SCIENCE:***

1. TS=(“Omega-3 Fatty Acid” OR “n3 Oil” OR “n3 Fatty Acid” OR “n3 PUFA” OR “n3 Polyunsaturated Fatty Acid” OR “N 3 Fatty Acid” OR “n 3 Polyunsaturated Fatty Acid” OR “Fish Oil” OR “Fish Liver Oils” OR “Linolenate” OR “alpha Linolenic Acid” OR “Linolenic Acid” OR “orator” OR “lovada” OR “EPA” OR “eicosapentaenoate-lipoate” OR “eicosapentaenoate-lipoate” OR “EPA-TA” OR “EPA-LA” OR “IPE” OR “valbazen” OR “EPA-TA” OR “EPA-LA” OR “omega-3 ethyl ester 90” OR “P-OM3 adjunct” OR “ethyl-EPA” OR “ethyl eicosapentaenoate” OR “ethyl icosapentaenoate” OR “ethyl eicosapentaenoic acid” OR “icosapent ethyl” OR “epadol” OR “AMR101” OR “viscera" OR “Docosahexaenoic Acid” OR “DHA” OR “Omegaven” OR “ostrya” OR “Icosapent” OR “Doconexent” OR “Omega-3-carboxylic acids” OR “Resolvin E1” OR “Icosapent ethyl” OR “Icosapent”)
2. TS=(“COVID 19” OR “SARS-CoV-2 Infection” OR “2019 Novel Coronavirus Disease” OR “2019 Novel Coronavirus Infection” OR “COVID 19 Virus Infection” OR “Coronavirus Disease 2019” OR “Severe Acute Respiratory Syndrome Coronavirus 2 Infection” OR “SARS Coronavirus 2 Infection” OR “COVID 19 Virus Disease” OR “2019 nCoV Infection” OR “COVID-19 Pandemics”)
3. #1 AND #2

*Date of search: 10 March 2022*

*Search results: 174*

*No limitations applied*

***SCOPUS :***

1. ALL (“Omega-3 Fatty Acid” OR “n3 Oil” OR “n3 Fatty Acid” OR “n3 PUFA” OR “n3 Polyunsaturated Fatty Acid” OR “N 3 Fatty Acid” OR “n 3 Polyunsaturated Fatty Acid” OR “Fish Oil” OR “Fish Liver Oils” OR “Linolenate” OR “alpha Linolenic Acid” OR “Linolenic Acid” OR “orator” OR “lovada” OR “EPA” OR “eicosapentaenoate-lipoate” OR “eicosapentaenoate-lipoate” OR “EPA-TA” OR “EPA-LA” OR “IPE” OR “valbazen” OR “EPA-TA” OR “EPA-LA” OR “omega-3 ethyl ester 90” OR “P-OM3 adjunct” OR “ethyl-EPA” OR “ethyl eicosapentaenoate” OR “ethyl icosapentaenoate” OR “ethyl eicosapentaenoic acid” OR “icosapent ethyl” OR “epadol” OR “AMR101” OR “viscera" OR “Docosahexaenoic Acid” OR “DHA” OR “Omegaven” OR “ostrya” OR “Icosapent” OR “Doconexent” OR “Omega-3-carboxylic acids” OR “Resolvin E1” OR “Icosapent ethyl” OR “Icosapent”)
2. ALL (“COVID 19” OR “SARS-CoV-2 Infection” OR “2019 Novel Coronavirus Disease” OR “2019 Novel Coronavirus Infection” OR “COVID 19 Virus Infection” OR “Coronavirus Disease 2019” OR “Severe Acute Respiratory Syndrome Coronavirus 2 Infection” OR “SARS Coronavirus 2 Infection” OR “COVID 19 Virus Disease” OR “2019 nCoV Infection” OR “COVID-19 Pandemics”)
3. #1 AND #2

*Date of search: 10 March 2022*

*Search results: 429*

*No limitations applied*

***EMBASE*** *:*

1. (Omega-3 Fatty Acid or n3 Oil or n3 Fatty Acid or n3 PUFA or n3 Polyunsaturated Fatty Acid or N 3 Fatty Acid or n 3 Polyunsaturated Fatty Acid or Fish Oil or Fish Liver Oils or Linolenate or alpha Linolenic Acid or Linolenic Acid or orator or lovada or EPA or eicosapentaenoate-lipoate or eicosapentaenoate-lipoate or EPA-LA or IPE or valbazen or EPA-TA or omega-3 ethyl ester 90 or P-OM3 adjunct or ethyl-EPA or ethyl eicosapentaenoate or ethyl icosapentaenoate or ethyl eicosapentaenoic acid or icosapent ethyl or epadol or AMR101 or viscera or Docosahexaenoic Acid or DHA or Omegaven or ostrya or Icosapent or Doconexent or Omega-3-carboxylic acids or Resolvin E1 or Icosapent ethyl or Icosapent )
2. (COVID 19 or SARS-CoV-2 Infection or 2019 Novel Coronavirus Disease or 2019 Novel Coronavirus Infection or COVID 19 Virus Infection or Coronavirus Disease 2019 or Severe Acute Respiratory Syndrome Coronavirus 2 Infection or SARS Coronavirus 2 Infection or COVID 19 Virus Disease or 2019 nCoV Infection or COVID-19 Pandemics)
3. #1 AND #2

*Date of search: 10 March 2022*

*Search results: 409*

*All fields searched*

*No limitations applied*
